# Effects of Wearing Different Personal Equipment on Force Distribution at the Plantar Surface of the Foot

**DOI:** 10.1155/2013/827671

**Published:** 2013-05-16

**Authors:** Christoph Schulze, Tobias Lindner, Sandra Woitge, Susanne Finze, Wolfram Mittelmeier, Rainer Bader

**Affiliations:** ^1^Department of Orthopaedics, University of Rostock, Doberaner Straße 142, 18057 Rostock, Germany; ^2^Bundeswehr Institute of Sports Medicine, Dr.-Rau-Allee 32, 48231 Warendorf, Germany; ^3^Rostock Military Medical Center, Hohe Duene 30, 18119 Rostock, Germany

## Abstract

*Background*. The wearing of personal equipment can cause specific changes in muscle activity and posture. In the present study, we investigated the influence of differences in equipment related weight loading and load distribution on plantar pressure. In addition, we studied functional effects of wearing different equipment with a particular focus on relevant changes in foot shape. *Methods*. Static and dynamic pedobarography were performed on 31 male soldiers carrying increasing weights consisting of different items of equipment. *Results*. The pressure acting on the plantar surface of the foot increased with higher loading, both under static and dynamic conditions (*p* < 0.05). We observed an increase in the contact area (*p* < 0.05) and an influence of load distribution through different ways to carry the rifle. *Conclusions*. The wearing of heavier weights leads to an increase in plantar pressure and contact area. This may be caused by flattening of the transverse and longitudinal arches. The effects are more evident in subjects with flat feet deformities which seem to flatten at an earlier load condition with a greater amount compared to subjects with normal arches. Improving load distribution should be a main goal in the development of military equipment in order to prevent injuries or functional disorders of the lower extremity.

## 1. Background

Soldiers are subjected to exhausting physical activities during military operations and especially during basic training. As a result, functional problems and traumatic lesions of the musculoskeletal system are frequently seen in military populations [1]. Women are affected approximately twice as often as men, and the majority of injuries involve the lower extremities [1]. The risk of sustaining an injury is approximately four times higher in athletes and approximately ten times higher in soldiers undergoing basic training than in the general population [1, 2]. Injuries due to musculoskeletal overuse arising from running or load carrying are more common than other traumatic injuries [1, 3]. Apart from general risk factors such as smoking, level of physical activity, and age, there are a number of other risk factors influencing the occurrence of injuries. These factors include high-arched feet, limited dorsal extension of the ankle, and flat arches [4–6]. Functional changes in the foot also play a role, especially in the development of musculoskeletal disorders as well as training- and exercise-related injuries [7]. These changes can be detected with pedobarography. Using this method, Becker et al. identified different parameters of ankle instability [8]. Likewise, patellofemoral pain syndrome can also be associated with biomechanical dysfunction of the rolling movement of the foot [9]. These examples demonstrate that on the one hand pedobarography can effectively detect biomechanical abnormalities of the sole of the foot attributable to a variety of pain syndromes or injury patterns of the lower limb, knee, or pain involving the lumbar spine [10]. On the other hand, foot deformities can also lead to pain syndromes in, for example, the knee or back [11].

Previous studies have shown that military equipment and footwear lead to stress on the lower extremities of soldiers and can contribute to the development of musculoskeletal disorders [12, 13]. For example, connections between increased muscular activity of the musculus tibialis anterior and development of shin splints were assumed. Equipment influences body axis and plantar pressure [12, 14]. Due to this fact the objective of the present study was to assess to what degree military equipment influences the vertical pressure forces that act on the plantar surface of the foot by various levels of loading with heavy equipment and changed load distribution. This may be one reason for the occurrence of musculoskeletal disorders. Hence, we selected the methods of static and dynamic pedobarography to derive some recommendations in the development of new equipments in order to be more functional and comfortable in practical use. Furthermore, it was considered important to analyse whether relevant changes in foot shape, such as splay and flat feet, affect the ability of the transverse and longitudinal arches to compensate for the effects of increasing loads. The changes detected have to be evaluated and interpreted and should influence preventive procedures to strain in medical support for occurred pain syndromes in the foot, for example.

## 2. Material and Methods

### 2.1. Subjects

**Figure 1:**
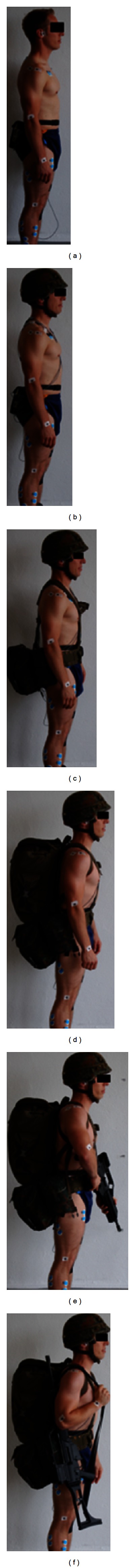
Load conditions: (a) no equipment, (b) helmet, (c) load-carrying equipment, (d) backpack, (e) rifle carried in front of the body, and (f) rifle slung over the shoulder.

Thirty-one male soldiers volunteered to participate in this experimental study. The study was approved by the Ethics Committee of the University of Rostock (no. A 2009 36). All participants gave their fully informed consent to take part in the study. They were aged between 20 and 53 (mean age 29) years, weighed between 62.5 and 112.0 (mean weight 81.7) kg, and had a height between 163 and 193 cm (mean value 177.7 cm) and a BMI between 21 and 34 kg/m^2^ (mean value 26.0). All participants had completed their basic training before taking part in the study. We performed a physical examination in order to assess the presence or absence of musculoskeletal disorders or deformities such as flat feet, and measure ankle, knee, hip, and shoulder ranges of motion. Discrimination of flat feet versus normal feet was performed according to the criteria of Whitman (valgus, abduction, and depression of the arch) by observers' decision [15]. As shown in Figure 1, the participants were asked to carry loads that were progressively increased. The musculoskeletal system was thus exposed to increasing stress. The various items of equipment and their weights are given in Table 1. 

### 2.2. Pedobarography

Pedobarography was performed in two setups, that is, static and dynamic measurements were carried out. During static pedobarography, the soldiers were asked to stand on the pressure-sensitive mat barefoot with both feet and carry increasing loads that consisted of a number of military equipment items (Figure 1). When the participants assumed a stable standing position, measurements were performed.

During dynamic pedobarography, the participants were asked to complete a 10-meter walkway barefoot at a steady pace with approximately similar velocity under the same changing load conditions like during the static pedobarography. During each walk, the loads were progressively increased. At a distance of approximately seven meters, the subjects walked over a pressure-sensitive mat (HR Mat Clinical 6.11; Tekscan, South Boston, MA, United States). We instructed them to continue to walk past the mat for approximately two meters in order to prevent them from discontinuing the walk immediately after the pressure measurement and thus avoid any effects of discontinuation of the movement on the measurements. Right and left feet were tested separately five times. Subject's walking speed could have influence on the measurement of force and pressure. In our setup there was no particular detection of walking speed during the test. This is a limitation of this study. To prove that there was no bias due to differences in walking speed, we measured the rolling velocity of the foot for each trial using the pressure-sensitive mat. Foot length and time of foot contact during stance phase were analysed to calculate rolling velocity.  Table 2 shows the results for each setup. There were no significant differences (average foot rolling velocity of 0.4 m·s^−1^); thus, there may be no relevant bias in the results based on walking speed variations. 

In both cases (static and dynamic investigations) we measured the contact area between the foot and the pressure-sensitive mat. The mat that was used in this study was 447 mm long, 487 mm wide, and 5.7 mm thick. It contained 8448 resistance sensing elements evenly distributed over the entire surface of the mat (4 sensors per cm²). All measurements were performed at a scan rate of 185 Hz. Data were analysed using appropriate software (HR Mat Clinical 6.11 System; Tekscan, South Boston, MA, United States). The following parameters were assessed: foot contact area (*A*), mean foot contact pressure at maximum foot contact (*P*), and maximum local contact pressure (*P*_max⁡_). During dynamic measurements stance phases were detected according to Perry's definition to characteristic footprint and pressure changes by observer's decision [16]. Peak values of each phase were detected and included in statistical processing. Heel Strike is the phase when the foot touches the ground (first possible measurement). Toe-off is the phase before the digitus 1 leaves the ground (last possible measurement). Midstance is the phase when maximum contact area of the foot was measured. These phases were identified by the specific character of the foot form on the monitor and the time flow by decision of the observer. We detected length and width of the longitudinal and transversal arch by analyzing the footprint in combination with anatomical facts. Therefore, heel and the head of metatarsal 1 were identified in the digital footprint by one observer. The maximum length between the dorsal end of the heel and the ventral end of the head of metatarsal 1 was measured as a footprint parameter for the longitudinal arch. To detect width, the heads of metatarsal 1 and 5 were identified in the digital footprint, and the distance between the lateral frontier of metatarsal 5 and the medial frontier of metatarsal 1 was measured by one observer. As a limitation we have to mention that intraobserver reproducibility was not analysed. 

### 2.3. Statistical Methods

Descriptive statistics (means, standard deviations, minimum, and maximum values) were calculated for each data set. Every subject carried progressively increasing loads consisting of various items of military equipment (Table 1). Thus, data were analysed using the Friedman test and the Wilcoxon test. It should be noted that the measurements provided subject-specific results. Determination of significant differences between the groups normal feet and flat feet was realized by the Kruskal-Wallis test (KW) and the Mann-Whitney *U* test (MW), respectively, as nonparametric test for comparison of independent samples. All *p* values were two-tailed, and a *p* value < 0.05 was considered significant. Data were stored and analysed using SPSS 15.0 software (SPSS Inc., Chicago, IL, USA). All *p* values of significant results are presented in Tables 3 to 4. 

## 3. Results

### 3.1. Dynamic Pedobarography

During initial ground contact, there was a small but significant increase in contact pressure at higher loads, that is, load-carrying equipment (*p* = 0.007), rifle compared to control (*p* = 0.007). The results for contact area did not show significant differences under the various load-carrying conditions during initial ground contact (Table 5). Moreover, data of the dynamic pedobarography giving an impression of the magnitude of the differences during midstance at the moment of maximum foot contact were demonstrated (Table 6). The largest differences occurred at this stage of the gait cycle under the various loading conditions. Contact pressures increased significantly with higher loads during midstance (all load conditions compared with each other except helmet versus control; *p* < 0.001). There was a particularly marked increase when the subjects carried a backpack (*p* < 0.001). In addition, total contact pressure was influenced by the way the rifle was carried. The values increased when the rifle was slung over the shoulder compared with when the rifle was carried in front of the body (*p* = 0.011).

During midstance, the contact area increased significantly at higher loads (*p* = 0.006). The most marked increase was again noted when the soldiers carried a backpack (*p* < 0.001). Rifle wearing had no significant influence (*p* = 0.236). The maximum contact pressure levels changed in the same way as the aforementioned parameter. Both parameters, contact pressure and contact area, also increased significantly during the final stage of the stance phase at higher loads (*p* < 0.001).

### 3.2. Static Pedobarography

Table 7 shows the effects of increased loading under static conditions, that is, when the subjects carried various loads in a standing position. In this condition, the mean bilateral contact pressure that acts on the plantar surface of the foot and contact area of the foot increased with higher loads (every load condition compared to control; *p* < 0.001). A particularly marked increase was noted when the subjects carried a backpack and a rifle (*p* < 0.001). In the standing position, a significant increase in the mean contact pressure acting on both foot soles was also noted when the rifle was carried over the right shoulder compared to the setup rifle in front of the body (*p* = 0.047). In particular, when the soldiers carried the rifle over the right shoulder, stress on the right foot increased significantly (71 kPa to 75 kPa; *p* = 0.001). Bilateral mean peak local pressure levels increased significantly depending on the load carried (*p* < 0.001). 

The pressure plate enabled identification of the position of a subject's centre of mass in the transverse plane. Only when the rifle was carried over the shoulder and not in front of the body did the centre of mass shift to the right. Neither load weight nor rifle wearing had a significant effect on the position of the centre of mass in the ventral direction.

**Figure 2:**
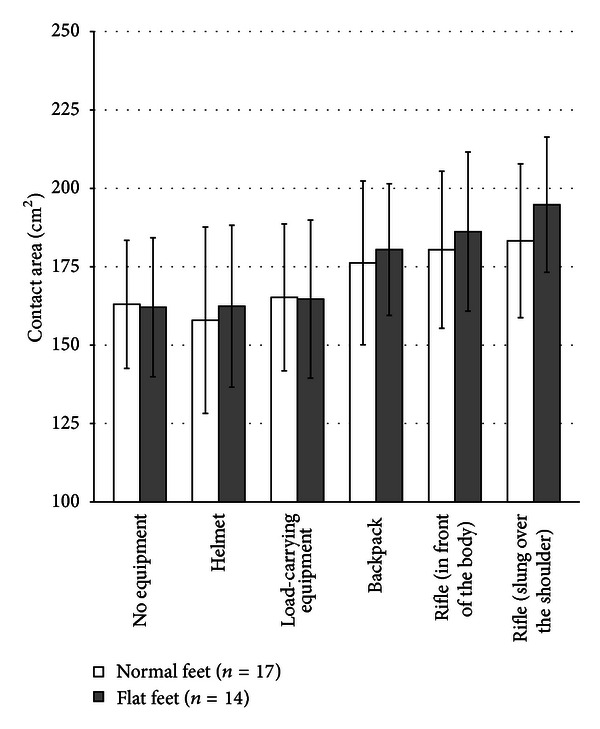
Contact area of both feet in cm^2^. Comparison of subjects with flat feet and subjects with normal feet as detected under static conditions.

Increasing maximum foot length, foot width, and contact area were detected with increasing loads (Table 7). Whereas the differences in foot length and width were not significant (*p* = 0.67), the contact area increased significantly with higher loads (*p* < 0.001). Rifle wearing as a marker for the influence of load distribution had a significant effect on the contact area. Wearing the rifle slung over the right shoulder led to an increased contact area compared to wearing rifle in front of the body (Table 7; *p* = 0.001). Soldiers with splay and flat feet, however, showed larger contact areas and higher increases in contact areas, but there was not enough power to demonstrate significance of that relevant fact (*p* = 0.053) (Figure 2). 

## 4. Discussion

As a result of their daily activities and training, soldiers are more affected by overuse and other injuries of the musculoskeletal system than the general population [1]. A variety of techniques are available for the detection of different load patterns. Pedobarography is a method for analysing the distribution of pressures and forces and thus allows specific changes in foot biomechanics and gait to be identified [8, 10]. In the present study, we could show that the wearing of greater weights and thus an increase in load led to changes in the distribution of pressures and forces on the sole of the foot. Our results from static and dynamic pedobarography and of the footprint measurements revealed that foot arch stability can also depend on the load to be carried, in so far as the longitudinal and transverse arches may tend to flatten when they are no longer able to compensate for the load. This has to be seen in the light of the methodological limitation that the influence of soft-tissue reactions cannot be clearly separated from arch flattening of the foot by detecting the contact area. Observations of other authors with athletes completing different athletic tasks support our findings [17, 18]. Furthermore, our findings suggest an increase in the contact area is associated with an increase in the distance from the heel to the first metatarsal caput and an increase in the distance from the first to the fifth metatarsal caput. The larger distances can be explained by a flattening of the transverse arch. This is supported by the findings of Queen et al. (2009), who detected increasing forces and contact areas as a consequence of strain to normal and flattened feet during athletic tasks [17]. 

Chen and Gielo-Perczak (2011) demonstrated an arch drop when the foot was statically loaded. However, they did not detect an influence on ground reaction forces [19]. A correlation between lower arch posture and greater pressure on the plantar surface was found by Jonely et al. (2011) [20]. By contrast, our study revealed a significant effect of the wearing of military equipment on vertical pressure. Not all subjects, however, showed significant changes in measurement parameters under the initial conditions when they carried moderate loads that consisted of a helmet and load-carrying equipment. This effect was detected under static conditions and especially under dynamic conditions during midstance. It could be explained by the change in load distribution in the presence of an additional horizontal force component that was not assessed. This could be proved using a three-component force plate, which was not available for the tests carried out. The one-dimensional pressure plate enabled calculation of the body's centre of mass on the basis of the distribution of pressures and forces. A change in the centre of mass was noted only in the transverse plane, when the rifle was slung over the shoulder and caused a shift of the centre of mass to the right. A displacement in the ventral or dorsal directions did not occur although a significant ventral inclination was observed. 

When analysing the static images with increasing load, these showed a forward inclination of the trunk in the sagittal plane and no change in inclination in the frontal plane, in accordance with the findings reported by Attwells et al. [12]. Despite a change in the body axis, the body is obviously able to maintain the body's centre of mass in a stable position in the sagittal plane through adaptation and the use of muscle force. Likewise, it is obviously able to compensate for an imbalance in the frontal plane through the use of muscle force, in order to prevent a displacement of the body axis. As described above, a change in the frontal plane was associated with wearing a rifle over the shoulder and resulted from an increase in the pressure and force acting on the sole of the foot. The detected changes due to carrying of the rifle in different ways indicate the importance of load distribution. In other investigations the response of trunk muscles, that is, M. trapezius and M. pectoralis major, also showed effects depending on load distribution [14]. Carrying loads ventral in front of the body led to significant reduction of muscular activity [21]. Our results support this fact so far, that with equal loads (rifle in front of the body and slung over the shoulder) the plantar pressure increased when the rifle was carried over the right shoulder. In addition, an imbalance between right and left foot was observed. Intelligent load-carrying systems with a ventral or dorsal possibility to wear loads balanced and close to the body centre could be a central issue in future developments of military equipment.

In comparison to soldiers without foot deformity, subjects with clinically detected flat feet deformities showed earlier and more marked flattening of the arches, which was reflected by a larger contact area. When the subjects slung their rifles over the shoulder, soldiers with splay and flat feet and improper arches showed an increasing contact area compared to subjects with normal feet (Figure 2). Due to the methodological limitation that separate evaluation of changes in the bony configuration of the foot arch is unfeasible because the influence of soft-tissue reactions cannot be clearly distinguished from arch flattening of the foot by detecting the contact area. However, the increasing contact area can be seen as a complex reaction to strain where flattening of the arch and soft-tissue reactions are included [18]. Hence, insoles should be taken into consideration to optimise the distribution of forces and pressures, as well as for prevention of injuries or functional disorders involving the feet, knee, and hip joint or lumbar spine. As shown by several authors, high local pressure at the plantar surface, discomfort, and pain can be reduced by wearing insoles [11, 22, 23]. Hence, a proactive treatment with insoles for soldiers with foot deformities should be considered. Furthermore, the present study points out the dependency on load wearing, for example, the position of the rifle; this needs to be taken into account for future development of carrying equipment. In this context, a limitation of this study is the fact that only male soldiers could be examined. 

In summary, we were able to show specific changes in foot contact area and contact pressure related to different military equipment. Because it is difficult to reduce the weight of the personal equipment that has to be worn during military operations the kind of load wearing (load distribution) is an important factor for future development of military equipment. 

## Figures and Tables

**Table 1:** Equipment items and weight.

Equipment item	Weight
Helmet	1.5 kg
Load-carrying equipment	1 kg
Backpack	15 kg
Rifle (G 36)	3.63 kg

**Table 2:** Rolling velocity during pedobarography.

State of burden	Control	Helmet	Load-carrying strap	Backpack	Rifle in front	Rifle slung over the shoulder
Rolling velocity	0.41 m/s	0.41 m/s	0.41 m/s	0.39 m/s	0.40 m/s	0.39 m/s
Standard deviation	0.03	0.04	0.04	0.03	0.03	0.03

**Table 3:** Calculated *p* values from Wilcoxon test after pairwise comparison of changes of foot contact area during static (*A* stat) and dynamic (*A* dyn) pedobarography. Significant results were highlighted with*.

		Control		Helmet		Plus load-carrying equipment		Plus backpack		Plus rifle (in front of the body)	
		Cervical spine	Body axis	Cervical spine	Body axis	Cervical spine	Body axis	Cervical spine	Body axis	Cervical spine	Body axis
Helmet	Cervical spine	*p* = 0.009*									
	Body axis		*p* = 0.112								

Plus load-carrying equipment	Cervical spine	*p* = 0.010*		*p* = 0.289							
	Body axis		*p* = 0.199		*p* = 0.347						

Plus backpack	Cervical spine	*p* < 0.001*		*p* < 0.001*		*p* < 0.001*					
	Body axis		*p* < 0.001*		*p* < 0.001*		*p* < 0.001*				

Plus rifle (in front of the body)	Cervical spine	*p* < 0.001*		*p* < 0.001*		*p*< 0.001*		*p* = 0.007*			
	Body axis		*p* < 0.001*		*p* < 0.001*		*p* < 0.001*		*p* = 0.002*		

Plus rifle (slung over the shoulder)	Cervical spine	*p* < 0.001*		*p* < 0.001*		*p* < 0.001*		*p* = 0.003*		*p* = 0.765	
	Body axis		*p* < 0.001*		*p* < 0.001*		*p* < 0.001*		*p* = 0.147		*p* = 0.056

**Table 4:** Calculated *p* values from Wilcoxon test after pairwise comparison of changes in foot contact pressure under static (*P* stat) and dynamic (*P* dyn) conditions and maximum contact pressure (*P* max dyn). Significant results were highlighted with*.

		Control			Helmet			Plus load-carrying equipment			Plus backpack			Plus rifle (in front of the body)		
		*P* stat	*P *dyn	*P *max dyn	*P* stat	*P *dyn	*P* max dyn	*P *stat	*P* dyn	*P *max dyn	*P* stat	*P *dyn	*P* max dyn	*P* stat	*P *dyn	*P* max dyn
Helmet	*P *stat	*p* < 0.001*														
	*P *dyn		*p = *0.620													
	*P* max dyn			*p = *0.610												

Plus load-carrying equipment	*P *stat	*p* < 0.001*			*p = *0.019*											
	*P *dyn		*p = *0.001*			*p = *0.002*										
	*P *max dyn			*p = *0.002*			*p = *0.013*									

Plus backpack	*P *stat	*p* < 0.001*			*p< *0.001*			*p < *0.001*								
	*P* dyn		*p < *0.001*			*p < *0.001*			*p < *0.001*							
	*P* max dyn			*p < *0.001*			*p < *0.001*			*p< *0.001*						

Plus rifle (in front of the body)	*P* stat	*p* < 0.001*			*p< *0.001*			*p < *0.001*			*p = *0.002*					
	*P* dyn		*p < *0.001*			*p < *0.001*			*p < *0.001*			*p = *0.776				
	*P* max dyn			*p < *0.001*			*p < *0.001*			*p < *0.001*			*p = *0.006*			

Plus rifle (slung over the shoulder)	*P* stat	*p* < 0.001*			*p < *0.001*			*p < *0.001*			*p = *0.001*			*p = *0.047*		
	*P* dyn		*p < *0.001*			*p < *0.001*			*p < *0.001*			*p < *0.001*			*p = *0.011*	
	*P* max dyn			*p < *0.001*			*p < *0.001*			*p < *0.001*			*p < *0.001*			*p = *0.001*

**Table 5:** Dynamic measurement results for contact area (*A*) and pressure (*P*) during initial ground contact.

	*P* in kPa	*A* in cm^2^
Control	260 ± 62	41 ± 7
Helmet	252 ± 82	38 ± 7
Plus load-carrying equipment	270 ± 48	40 ± 6
Plus backpack	271 ± 68	41 ± 5
Plus rifle (in front of the body)	269 ± 68	40 ± 7
Plus rifle (slung over the shoulder)	279 ± 73	40 ± 7

**Table 6:** Dynamic measurement results for contact area (*A*), pressure (*P*), and maximum pressure (*P*_max⁡_) during midstance.

	*P* in kPa	*A* in cm^2^	*P*_max⁡_ in kPa
Control	120 ± 18	179 ± 23	337 ± 88
Helmet	120 ± 21	179 ± 23	339 ± 90
Plus load-carrying equipment	129 ± 17	183 ± 23	385 ± 112
Plus backpack	148 ± 22	193 ± 22	462 ± 148
Plus rifle (in front of the body)	149 ± 22	198 ± 23	443 ± 116
Plus rifle (slung over the shoulder)	155 ± 23	196 ± 24	483 ± 140

**Table 7:** Static measurement results for contact area (*A*), pressure (*P*), distance from the first to the fifth metatarsal caput (MTP I–V), and distance from the heel to the first metatarsal caput.

	*P* in kPa	*A* in cm^2^	ΔMTP I–V in cm	ΔAnkle-MTP I in cm
Control	111 ± 13	162 ± 21	9.1 ± 0.7	20.4 ± 0.9
Helmet	119 ± 20	163 ± 25	9.1 ± 0.8	20.4 ± 1.2
Plus load-carrying equipment	121 ± 17	162 ± 23	9.1 ± 0.6	20.5 ± 0.9
Plus backpack	138 ± 21	178 ± 23	9.2 ± 0.7	20.6 ± 1.0
Plus rifle (in front of the body)	145 ± 18	183 ± 25	9.3 ± 0.7	20.6 ± 0.9
Plus rifle (slung over the shoulder	147 ± 19	188 ± 23	9.3 ± 0.7	20.7 ± 0.9
